# Heart rate variability: a multidimensional perspective from physiological marker to brain-heart axis disorders prediction

**DOI:** 10.3389/fcvm.2025.1630668

**Published:** 2025-11-06

**Authors:** Shujuan Liu, Yunyi Cui, Meihong Chen

**Affiliations:** 1 Baoding First Central Hospital, Baoding, Hebei Province, China; 2 Department of Oral and Maxillofacial Surgery, School and Hospital of Stomatology, Hebei Medical University & Hebei Key Laboratory of Stomatology & Hebei Clinical Research Center for Oral Diseases, Shijiazhuang, China

**Keywords:** heart rate variability, autonomic nervous system, sympathetic branch, parasympathetic branch, mixed branch, cardiovascular disease, mental health, obesity

## Abstract

Heart rate variability (HRV), a non-invasive measure of autonomic nervous system (ANS) activity and homeodynamics, has received much attention in recent years in the study of cardiovascular disease, mental health, and aging. Changes in HRV not only reflect an individual's ability to adapt to changes in the internal and external environment but also correlate with a wide range of pathological states, making it a powerful tool for predicting disease risk and assessing the efficacy of treatment. The aim of this review is to comprehensively analyze the role of HRV in different physiological and pathological contexts and explore its value as a potential biomarker. Initially, we review the basic concepts, measurements, and influencing factors of HRV, followed by an in-depth discussion of the relationship between HRV and cardiovascular disease, epilepsy, depression, aging, and inflammation. Special emphasis is placed on the role of HRV in assessing the health impact of obesity, nutrition, and lifestyle. Additionally, we explore the use of HRV in clinical practice, including its potential in predicting disease, guiding treatment, and evaluating the effects of interventions. Ultimately, we suggest future research directions, including the promise of HRV in individualized medicine and health monitoring. While HRV holds promise as a non-invasive, trans-diagnostic biomarker, current evidence remains preliminary and largely associative. Its clinical utility for personalized medicine or routine risk prediction requires standardized acquisition protocols, external validation, and causal inference studies before implementation into decision-making algorithms. By synthesizing multiple studies through the lens of brain - heart axis (BHA) integrity, we propose that HRV metrics serve as a quantifiable, trans-diagnostic proxy for mapping the measurement, mechanistic, and translational axes of brain - heart dysfunction.

## Introduction

HRV refers to the variations in the time intervals between consecutive heartbeats (1). It is a complex physiological phenomenon that reflects the dynamic regulation of the cardiovascular system by the ANS, which consists of sympathetic and parasympathetic nervous systems. The sympathetic nervous system generally increases heart rate and cardiac output, while the parasympathetic nervous system has the opposite effect, slowing down the heart rate. These two systems work in a coordinated manner to maintain cardiovascular homeodynamics (dynamic, scale-free stability rather than static equilibrium). Contemporary models emphasize that beat-to-beat variability emerges from the interaction of central autonomic networks with intrinsic sino-atrial ion-channel kinetics, baroreceptor feedback, respiratory-cardiac coupling and rapidly shifting hormonal milieus (2, 3).

One hypothesis posits that there is an additional fourth control level in cardiovascular regulation, where the amplitude of low-frequency HRV (LF-HRV) serves as a reference input for the neural cardiovascular center (1). This center then responds to maintaining LF-HRV around a certain level. For example, the absence of LF-HRV during artificial cardiac pacing may be associated with pacemaker syndrome, despite seemingly normal cardiovascular performance. This suggests that HRV plays a crucial role in the overall regulation of the cardiovascular system and that disruptions in this regulation could lead to various morbidities.

HRV research has far-reaching importance and a wide scope of application. It has been associated with a range of health conditions, making it a valuable biomarker in both clinical and research settings. In terms of disease prediction, lower HRV has been linked to an increased risk of mortality, including all-cause and cardiac mortality. A meta-analysis involving 32 studies and two individual participant datasets with 38,008 participants found that lower HRV parameter values were significant predictors of higher mortality across different ages, sexes, continents, populations, and recording lengths (4).

HRV biofeedback has also shown promise in various applications. For example, in patients with coronary artery disease, HRV biofeedback could reduce ANS reactivity during anger events and increase ANS recovery after such events (5). This indicates its potential as a therapeutic tool in cardiac rehabilitation. Additionally, HRV measurement could be used to monitor the effects of lifestyle interventions, such as exercise and dietary changes, on autonomic function, providing insights into the effectiveness of these interventions in promoting health and preventing disease.

The BHA is a bidirectional neuro-humoral communication network in which cortical, limbic and brain-stem centers dynamically modulate cardiac autonomic tone, while afferent cardiac signals in turn influence cerebral activity and emotional regulation. Disruption of this loop - via chronic stress, systemic inflammation, neurodegeneration or metabolic dysautonomia - has been implicated in both primary cardiac disorders (e.g., myocardial infarction, heart failure) and primary neurological/psychiatric disorders (e.g., epilepsy, depression, Alzheimer's disease). Because HRV is a non-invasive, real-time read-out of vagal and sympathetic outputs, it provides an integrative window into BHA integrity. Therefore, alterations in HRV may not merely reflect isolated cardiac risk, but rather signal broader BHA disturbances that predispose to or perpetuate multi-system disease. Acknowledging the BHA explicitly reframes HRV from a peripheral cardiovascular metric to a central nervous system–cardiovascular coupling biomarker. The neuroanatomical substrates of BHA include cortical (e.g., prefrontal cortex), limbic (e.g., amygdala), and brainstem (e.g., nucleus tractus solitarius) regions, as detailed by Thayer & Lane (6) and Critchley & Harrison (7).

The aim of this review is to provide a comprehensive and critical report on the factors influencing HRV measurement, its role in relation to cardiovascular disease, neurology, and lifestyle assessment, and to elaborate on its status and outlook. By elaborating on these issues, it is hoped that a deeper understanding of the complexity of HRV will be gained, thus enabling physicians to better apply HRV in monitoring health and diagnosing disease in the clinical setting.

### Conceptual framework

a. Define “BHA integrity” (top-down cortical inhibition ↔ vagal modulation ↔ peripheral feedback).
b. Explain why HRV maps onto three axes:

Measurement axis: which metric captures which limb of the BHA.

Mechanistic axis: how BHA disruption (stress, inflammation, neurodegeneration) translates into specific HRV signatures.

Translational/predictive axis: how HRV-guided interventions restore BHA integrity and improve dual cardiac - neurologic outcomes.

c. Set out three testable claims that the review will evaluate:

Reduced HRV consistently marks BHA disruption across cardiac, metabolic and neuro-psychiatric diseases.

Non-linear HRV parameters are the earliest to decline when central autonomic network integrity is lost.

HRV biofeedback/lifestyle interventions that raise vagal tone simultaneously improve cardiac and neurologic endpoints via BHA restoration.

### Evidence synthesis and quality grading strategy

To address methodological heterogeneity, we adopted a three-tier evidence-grading system adapted from the GRADE working group.

Tier 1 (High): Prospective cohorts ≥500 participants or meta-analyses with ≥3 studies, adjusted effect sizes (HR, *β*) and 95% CI reported.

Tier 2 (Moderate): Cross-sectional or case–control studies ≥100 participants with effect sizes and 95% CI.

Tier 3 (Low): Pilot RCTs < 50 participants or conference abstracts without adjustment or CI; findings are hypothesis-generating only.

Throughout Results, each cited outcome is tagged with its tier (T1 - T3) and the numeric effect size plus 95% CI when available. When original papers did not supply CIs, we computed them from the published raw counts or standard errors. This tiering is explicitly reported in every summary sentence (e.g., “T1 evidence: HR = 1.41, 95% CI 1.16–1.72”).

### HRV & ANS introductory

HRV arises from the dynamic interplay between sympathetic and parasympathetic efferent signals that continuously fine-tune sinoatrial node pacemaker activity; thus, every HRV metric could ultimately be mapped to one or both autonomic limbs (8). Post-ganglionic sympathetic fibers release noradrenaline, accelerating heart rate and shifting spectral power toward the low-frequency (LF) band (≈ 0.04–0.15 Hz). Heightened sympathetic drive-observed in heart failure, acute myocardial infarction, and psychosocial stress-manifests as reduced standard deviation of normal-to-normal intervals (SDNN), elevated low frequency power/high frequency power (LF/HF) ratio, and blunted non-linear complexity (9). The vagus nerve releases acetylcholine, inducing brady-cardia and high-frequency oscillations (≈ 0.15–0.40 Hz). Parasympathetic withdrawal, quantified by decreases in root mean square of successive differences (RMSSD), percentage of pairs of adjacent NN intervals differing by more than 50 ms (pNN50) and high frequency (HF) power, has been documented in major depression and during the post-ictal phase of epilepsy (10). Most real-world recordings reflect concurrent sympathetic and parasympathetic modulation. Global indices such as SDNN and SD2 (Poincaré long-axis) capture this combined influence, while non-linear metrics including SD2/SD1 ratio and DFA-α1 quantify system complexity. Advancing age progressively attenuates these mixed signals, underscoring HRV's role as a biomarker of age-related decline in autonomic function (11).

Beyond neural control, HCN4 and Ca_V_1.3 channels set the intrinsic pacemaker slope, while rapid β-adrenergic phosphorylation and slower genomic thyroid-hormone effects modulate channel gating and thus contribute to both short- and long-term HRV patterns (3).

Low-frequency power (LF, 0.04–0.15 Hz) and the LF/HF ratio have been repeatedly shown to reflect mixed sympathetic - parasympathetic modulation rather than a pure “sympathetic” index, especially when respiratory parameters and baroreflex gain are not held constant (12–14). Under paced breathing at 0.1 Hz, LF oscillations are largely baroreflex-mediated and can be vagally amplified; conversely, during orthostatic or isometric stress with unchanged respiratory rate, an LF increase may indicate sympathetic predominance. Therefore, we follow the 1996 Task Force consensus: autonomic labels should not be assigned to LF or LF/HF without concurrent physiological context (controlled breathing, posture standardization, or baroreflex testing).

#### Measurement techniques and standardization of HRV

Measurement of HRV involves several techniques, and standardization is crucial for accurate and comparable results. Electrocardiogram (ECG) is the gold-standard method for obtaining the R-R intervals, which are used to calculate HRV indices (15). However, with the advancement of technology, smartphone applications using photoplethysmography (PPG) have also been developed to measure HRV. For example, Moya-Ramon et al. (2022) validated Elite HRV (chest-strap ECG) and Welltory (PPG) against 12-lead ECG-derived RMSSD, low frequency (LF) and HF in 30 elite cyclists (supine RMSSD 45 ± 12 ms, seated 38 ± 11 ms) (16). These applications showed no differences compared to ECG in supine and seated positions and had very strong to almost perfect correlation levels (*r* = 0.77–0.94). Commercial smartphone/PPG apps often lack raw-data transparency, impeding manual ectopy editing and arrhythmia screening. Consequently, artefacts can inflate RMSSD or HF power by >30%, yielding artifactually “high” yet clinically meaningless variability (17). Until open raw-data access and validated beat-classification algorithms become standard, correlations of *r* ≈ 0.77–0.94 against ECG should be regarded as provisional rather than “near perfect”.

PPG-derived inter-beat-intervals are intrinsically affected by pulse transit time variability (PTTV) and arterial compliance changes across postures, ambient temperature, and exercise intensities. During active standing or walking, PTTV can introduce ±20–40 ms beat-to-beat dispersion that is not of neural origin, leading to systematic over-estimation of LF power and under-estimation of vagal indices (RMSSD, HF). Consequently, LF/HF ratios from wrist-PPG can differ by >1.0 compared with simultaneous ECG in the same individual (unpublished observations, *n* = 24). Sampling jitters are another under-reported limitation. Most consumer wearables buffer optical data at 20–50 Hz; sub-optimal peak-detection algorithms can produce epoch-dependent timing errors of 5–15 ms, inflating SDNN by 5%–10% in 5 min recordings and corrupting entropy measures that are scale-dependent. In addition, PPG amplitude loss during ectopic beats or premature contractions frequently escapes the device's internal artefact flag, resulting in missed ectopy or false-positive IBI insertion. Therefore, PPG-based HRV cannot be equated with ECG metrics until the following minimal validation protocol is satisfied (summarised in Table 1). Studies that skip any of these steps should be graded “Tier-3/low certainty” when cited.

**Table 1:** Validation checklist.

Minimum validation checklist for wearable HRV devices intended for clinical research
1. Head-to-head comparison with 12-lead or 3-lead ECG (≥256 Hz) in ≥20 participants.
2. Simultaneous recordings in three postures (supine, seated, standing) and at least two everyday activities (e.g., treadmill walking @ 4 km h^−^^1^, typing).
3. Manual editing concordance: blinded manual review of raw IBI series; report % of beats re-labelled and Bland - Altman limits of agreement for RMSSD and SDNN.
4. Ectopy-handling check device performance for PVC/PAC detection vs. ECG (sensitivity & specificity). If <90% sensitivity, apply offline correction and re-calculate HRV.
5. Public release of de-identified IBI files and code to allow third-party replication.

Standardization of HRV measurement is essential due to the influence of numerous factors on HRV values. A study investigated the reliability of short - term HRV measurements in different settings and positions, such as supine and standing, at home and in the laboratory (18). The environment was found to significantly impact standing HRV, with home measurements showing slightly lower variance compared to lab settings. This highlights the need for controlled conditions and consistent protocols in HRV monitoring and interpretation to ensure the accuracy and comparability of results across different studies and clinical applications (summarised in Table 2).

**Table 2:** Clinical decision.

Practical recommendation
Investigators should
(1) inspect raw inter-beat-interval series,
(2) apply published ectopy-detection rules ≥5% threshold,
(3) report both pre- and post-editing HRV values; failure to do so risks systematic over- or under-estimation.
(4) authors must report all 10 items above; failure to do so risks systematic bias and precludes meta-analysis.

Reporting checklist (adapted from Laborde et al. 2017 and Task Force 1996) (19, 20)

a. Recording duration: ≥5 min short-term (ultra-short ≤1 min only for RMSSD); 24 h for prognostic indices.
b. ECG sampling frequency: ≥256 Hz recommended; ≤500 Hz acceptable if hardware-limited.
c. Artifact/ectopy detection: automated algorithm + manual inspection; ≥5% ectopic beats → exclude segment or report pre- and post-editing values.
d. Interpolation method: cubic spline or Lomb-Scargle for gap ≤3 beats; >3 consecutive gaps → discard epoch.
e. Interpolation method: cubic spline or Lomb-Scargle for gap ≤3 beats; >3 consecutive gaps → discard epoch.
f. Posture: supine, seated, or standing; specify duration of habituation.
g. Time-of-day: report clock time and fasting/exercise status within preceding 3 h.
h. Medication log: substance, dose, time of last intake relative to recording; note sympatholytic, anticholinergics, *β*-blockers.
i. Environmental conditions: quiet, temperature 22–24°C; home vs. laboratory.
j. Data sharing: provide de-identified IBI series and codebook (e.g., PhysioNet-compatible format) to allow re-analysis.

#### Factors affecting HRV

Multiple factors could influence HRV, including age, sex, disease states, and lifestyle factors. Physical activity, orthostatic shifts, circadian rhythm of cortisol secretion, and fluctuating sex steroids further sculpt HRV through dynamic autonomic and direct electrophysiological actions (2). In patients with atrial septal defect (ASD), factors such as age, sex, defect diameter, heart rate, and diabetes were found to be associated with HRV indices (21). For example, in a study of 154 ASD patients who underwent transcatheter closure, age, sex, and defect size were among the factors that affected HRV. The SDNN and standard deviation of the average normal-to-normal intervals (SDANN), two HRV indices, were significantly higher after closure, and these indices had obvious correlations with right ventricular systolic pressure. Spontaneous breathing at 0.25 Hz (15 breaths min^−^^1^) can inflate HF power and depress LF, whereas 0.1 Hz breathing synchronizes with LF and artificially elevates the LF/HF ratio even when sympathetic output is unchanged (22).

Mental health conditions also play a role. In patients with schizophrenia and major depressive disorder, age-related variations in HRV were observed (23). Adults had reduced time-domain and nonlinear HRV compared to adolescents. Additionally, female subjects demonstrated lower time-domain HRV, LF/HF, and SD2 than males. Stress and negative affectivity could also impact HRV. In a study of patients with functional somatic syndromes, elevated negative affectivity and comorbid depression were associated with changes in HRV, although the results regarding the moderating role of HRV in endogenous pain modulation were inconclusive (24).

#### Medication effects on HRV

Pharmacological modulation of autonomic tone represents a major source of uncontrolled variance. β-blockers, non-dihydropyridine calcium-channel blockers, and centrally acting sympatholytics typically raise RMSSD and HF power, whereas tricyclic antidepressants, anticholinergics, and some antipsychotics reduce vagal indices. When baseline HRV is used for risk stratification, a complete medication history, including dose and timing, is therefore essential; failure to adjust for these agents can shift SDNN by >20 ms and LF/HF by >1 unit (25).

#### Non-linear parameters of HRV

Beyond linear metrics, non-linear HRV analyses capture the complexity and irregularity of the cardiac rhythm. Poincaré-derived indices (SD1, SD2, SD2/SD1) and entropy-based measures (ApEn, SampEn) provide additional insight into sympatho-vagal balance (26), while detrended fluctuation analysis (DFA)-α1/α2 and correlation dimension (D2) quantify the fractal properties and system complexity that are often blunted in autonomic dysfunction (27). Specific information is as follows.

i. Poincaré plot geometry

SD1 (short-axis dispersion) is dominated by parasympathetic modulation and strongly correlates with RMSSD.

SD2 (long-axis dispersion) reflects the joint influence of sympathetic and parasympathetic limbs, paralleling SDNN and LF power.

SD2/SD1 ratio provides a geometric analogue of the LF/HF ratio and tracks sympatho-vagal balance shifts.

ii. Entropy measures

Approximate Entropy (ApEn) and Sample Entropy (SampEn) quantify signal regularity; increases in both indices are seen during mental stress and are associated with reduced autonomic complexity rather than pure vagal withdrawal.

iii. Detrended Fluctuation Analysis (DFA)

α1 (short-term scaling exponent) is modulated by both autonomic branches, whereas α2 (long-term exponent) rises with relative shift toward sympathetic predominance and reduced vagal activity.

iv. Correlation Dimension (D2)

A lower D2 indicates loss of non-linear complexity and has been linked to impaired parasympathetic modulation in HIV-positive patients on antiretroviral therapy.

Non-linear parameters of HRV have emerged as critical tools in understanding the complex dynamics of cardiac autonomic regulation, particularly under varying physiological and psychological conditions. These parameters offer insights beyond traditional linear metrics, capturing the intricate interplay between sympathetic and parasympathetic nervous systems. The study of non-linear HRV parameters, such as correlation dimension, entropy, and DFA, has been pivotal in elucidating the autonomic responses to different stressors and interventions.

Research has demonstrated that non-linear HRV parameters are sensitive indicators of mental and physical stress. For instance, a study comparing HRV during paced breathing and mental arithmetic tasks found significant differences in non-linear parameters, highlighting their utility in distinguishing between relaxed and stressed states (26). Similarly, the impact of mental workload on HRV was investigated, revealing that mental tasks significantly reduce the complexity of HRV, as evidenced by a decrease in the correlation dimension (D2) (28). These findings underscore the potential of non-linear HRV metrics as reliable indicators of mental stress and workload.

In the context of exercise, non-linear HRV parameters have been shown to reflect the body's adaptive responses. During cycling exercise with varied cadence, non-linear dynamics of HRV, measured through DFA, indicated a decrease in complexity with increased exercise intensity, suggesting a shift from autonomic to non-autonomic control at higher intensities (29). This aligns with findings from another study that employed a novel non-linear model to characterize RR interval fluctuations during exercise and recovery, demonstrating the model's ability to provide precise assessments of autonomic function (30).

Furthermore, non-linear HRV parameters have been explored in clinical settings, offering insights into disease states and therapeutic interventions. In Parkinson's disease, non-linear HRV metrics have been used to assess autonomic function during dry immersion sessions, revealing compensatory mechanisms in cardiovascular regulation despite neurodegeneration (31). Additionally, non-linear HRV analysis has been employed to evaluate the safety and efficacy of treatments in frail elderly patients with secondary anemia, confirming the absence of cardiovascular risk associated with the intervention (32).

Overall, the integration of non-linear HRV parameters into research and clinical practice provides a more comprehensive understanding of autonomic regulation. These parameters not only enhance the assessment of physiological and psychological states but also offer valuable prognostic information in various health conditions. As research continues to evolve, the application of non-linear HRV metrics is likely to expand, further solidifying their role in advancing cardiovascular and autonomic health monitoring. All of these parameters have been inserted into the revised Table 3 with their corresponding ANS branches and physiological caveats.

**Table 3:** HRV components and their implicated autonomic nervous system branches.

Domain	Metric	Primary ANS branch	Physiological remarks/caveats
Time	SDNN	Sympathetic + Parasympathetic (mixed)	Global HRV; reflects combined autonomic modulation
Time	RMSSD	Parasympathetic	Fast beat-to-beat variation; vagally mediated
Time	pNN50	Parasympathetic	High-frequency RR-interval differences; sensitive to vagal tone
Frequency	LF (0.04–0.15 Hz)	Mixed (sympathetic slightly dominant)	Influenced by baroreflex, both limbs; posture & breathing affect interpretation
Frequency	HF (0.15–0.40 Hz)	Parasympathetic	Respiratory-linked vagal activity
Frequency	LF/HF ratio	Sympatho-vagal balance	Higher values indicate sympathetic dominance or reduced vagal tone
Non-linear	SD1 (Poincaré)	Parasympathetic	Geometric equivalent of RMSSD
Non-linear	SD2 (Poincaré)	Sympathetic + Parasympathetic	Correlates with long-term variability; related to SDNN
Non-linear	α1 (DFA)	Mixed	Short-term scaling exponent; influenced by both limbs
Non-linear	Entropy (SampEn, Rényi)	Mixed	Complexity indices; no single autonomic branch attribution

a. Short-term recordings (<5 min), body posture, breathing pattern, medications, age, and comorbidities could shift these associations. Always adjust these factors in clinical or research settings. b. Linear SDNN, RMSSD, LF and HF assume stationarity; DFA-α1, SampEn and SD1/SD2 do not, making them suitable for non-stationary heartbeat series. c. Medications (β-blockers, anti-arrhythmics, antidepressants, antipsychotics) and their dosing schedules can override the autonomic branch associations listed above; always record and, where possible, statistically adjust for these confounders. d. LF (and consequently LF/HF) lacks unique sympathetic specificity; interpretation requires simultaneous respiratory rate, posture, and baroreflex information. See main text for details and references.

#### Clinical maturity notes

At present, only linear time-domain metrics (SDNN, RMSSD) meet Tier-1 evidence thresholds for prognostic or diagnostic claims across multiple cohorts (see Evidence-grade summary). Non-linear indices (SampEn, DFA-α1, α2, D2) remain exploratory: between-study effect sizes vary >2-fold, reference ranges overlap substantially between health and disease, and no large-scale prospective data link them to hard clinical endpoints. Thus, entropy or fractal measures should be interpreted as hypothesis-generating unless replicated in ≥500-participant, adjusted, prospective cohorts. We explicitly priorities SDNN and RMSSD for all clinical statements in this review.

### Brain - heart axis: mechanistic mapping of HRV to neural circuits

Across two independent resting-state fMRI data-sets (total *n* = 156), higher resting RMSSD or HF power was consistently accompanied by stronger functional connectivity between the amygdala and the medial pre-frontal cortex (mPFC) as well as between the amygdala and the anterior cingulate cortex (ACC); these links remained significant after adjustment for age, sex and depression score (T1) (33). Wei et al. revealed that individual differences in HRV were linked to the coordinated microstructure of white-matter pathways connecting the prefrontal cortex with the amygdala: people exhibiting higher resting HRV showed greater structural covariance (thicker, more organized fibers) along these tracts, indicating that a stronger prefrontal-amygdala structural network may underpin the parasympathetic control of heart rate and emotion regulation (34). Using fMRI and simultaneous ECG while participants reappraised negative images, the authors found that trial-by-trial increases in high-frequency HRV tracked the strength of negative coupling between the amygdala and dorsolateral/dorsomedial prefrontal cortex; individuals with higher resting HRV showed both larger prefrontal down-regulation of amygdala activity and greater behavioral reduction of negative affect, indicating that flexible autonomic control and effective emotion regulation share a common prefrontal-amygdala functional circuit (35).

At the animal causation study, the vagus nerve - brainstem circuit regulates cytokine balance through specific neuronal subpopulations, directly influencing the inflammatory regulatory function of HRV. In mouse models, following LPS-induced inflammation via intraperitoneal injection, vagal TRPA1^+^ sensory neurons selectively respond to the anti-inflammatory cytokine IL-10, transmitting signals to the caudal nucleus of the solitary tract (cNST) in the brainstem. Activation of DBH^+^ neurons within the cNST significantly reduced proinflammatory factor (IL-1β) levels while elevating anti-inflammatory factor (IL-10) levels. Activation of this circuit increased survival rates to 90% in mice treated with a lethal dose of LPS (T3, validated through chemogenetic modulation, single-cell sequencing, and ablation experiments) (36). Further studies confirm that vagus nerve transection completely abolishes cNST's regulatory effect on inflammation, while ablation of DBH^+^ neurons reverses HRV-associated anti-inflammatory phenotypes. This establishes the neural-cytokine pathway as the core mechanism for HRV-mediated immune homeostasis (T3, based on bidirectional intervention experiments) (36).

A non-invasive human study found that transcutaneous auricular vagal nerve stimulation (taVNS) can enhance HRV metrics in a dose-dependent manner by targeting the auricular vagal nerve branch, with effects correlated to the stimulation site and EEG activity. Specifically, in a randomized controlled trial involving 13 healthy subjects, true stimulation point (concha) intervention resulted in over 30% increases in RMSSD and pNN50 from baseline, accompanied by enhanced frontal theta band activity. This oscillatory activity showed a positive correlation with HRV elevation; In contrast, stimulation at the control point (outside the tragus) only slightly increased SDNN and was associated with gamma-band activity in the frontotemporal region (T2, based on randomized controlled design and EEG-HRV synchrony analysis) (37). Furthermore, taVNS-induced HRV elevation sustainably improved autonomic balance, and frontal theta activity served as a biomarker predicting HRV regulation efficiency (T3, based on short-term intervention follow-up) (37).

Future research could focus on three directions: (1) Validating prefrontal-amygdala circuit dynamics in larger, diverse human cohorts (e.g., clinical populations with autonomic/emotion disorders) to confirm HRV-brain connectivity generalizability; (2) Exploring the vagus nerve-brainstem-cytokine pathway's translational potential - e.g., targeting TRPA1^+^/DBH^+^ neurons to modulate HRV and treat inflammation-related diseases; (3) Optimizing taVNS protocols (e.g., stimulation parameters, personalized site selection) using frontal theta as a real-time biomarker, and testing long-term taVNS effects on HRV, immune function, and emotional health in larger longitudinal studies. Additionally, integrating multi-modal tools (e.g., simultaneous fMRI-EEG-HRV, single-cell transcriptomics) could deepen understanding of HRV's neural-immune-emotional mechanisms, enabling more precise autonomic and therapeutic interventions.

### Sympathetic branch dysfunction

#### Measurement axis: HRV as a predictor of cardiovascular risk

HRV has shown promise as a predictor of cardiovascular risk. A meta - analysis of cohort studies found that lower HRV was associated with a higher risk of all-cause death and cardiovascular events in patients with cardiovascular disease (38). The pooled hazard ratio for all-cause death was 2.27 [95% confidence interval (CI): 1.72, 3.00], and for cardiovascular events was 1.41 (95% CI: 1.16, 1.72). In subgroup analyses, the association was significant for patients with acute myocardial infarction but not for those with heart failure in the case of all-cause death, and for patients with acute myocardial infarction and acute coronary syndrome but not for those with coronary artery disease and heart failure in the case of cardiovascular events.

Addleman et al. (Appl Psychophysiol Biofeedback 2025) synthesized 67 studies (2020–2024) and report moderate-quality evidence that reduced resting HRV-particularly SDNN < 70 ms or LF/HF > 2.5 - is associated with a 1.5- to 2.3-fold higher risk of major adverse cardiovascular events (MACE), while postoperative HRV decline could predict ICU cardiovascular complications 24–48 h in advance (39). In acute myocardial infarction, 24 h HRV indices (RMSSD, SDNN) are used for early risk stratification, with SDNN < 50 ms aiding ICD decision-making; Extremely elevated HRV-especially when driven by atrial fibrillation or frequent ectopy-can masquerade as “good” autonomic flexibility and must be distinguished from genuine vagal predominance (17). In chronic heart failure (NYHA II - III), six-week HRV-biofeedback training increased SDNN by 20–30 ms and improved 6-minute-walk distance and NT-proBNP (39). Among hypertensive patients, HRV biofeedback combined with antihypertensive medication lowered systolic BP by an additional 4–6 mmHg, although study sizes were small (*n* < 150). Heterogeneity remains high (*I*^2^ = 62%) due to inconsistent recording durations, frequency-band definitions, and inadequate adjustment for medications, circadian rhythm, and comorbidities (39). The authors conclude that HRV is a promising adjunct for early cardiovascular risk detection and monitoring therapeutic response but emphasize the need for standardized protocols and large multicenter RCTs to establish its clinical utility. Consistent with the finding, SDNN and RMSSD were also significantly decreased in patients with hypertension, suggesting increased sympathetic nervous activity (40).

In patients with hidradenitis suppurativa, an inflammatory skin disease associated with increased cardiovascular risk, HRV analysis has shown increased sympathetic activity, indicating a higher risk of cardiovascular disease (41). This suggests that HRV could be used to identify individuals at risk of cardiovascular complications even in the context of non-traditional cardiovascular risk factors.

#### Evidence-grade summary

- T1: Addleman et al. 2025 (67 studies, *n* = 38 008) - resting SDNN < 70 ms vs. ≥70 ms: MACE HR = 1.73, 95% CI 1.45–2.07 (ref. 28).
- T1: Fang et al. 2020 (meta-analysis, 32 cohorts, *n* = 35 042 CVD patients) – all cause mortality HR = 2.27, 95% CI 1.72–3.00; CV events HR = 1.41, 95% CI 1.16–1.72 (ref. 27).
- T2: He et al. 2024 (cross-sectional, *n* = 348 hypertension) - SDNN↓ 22 ms, Cohen's d = 0.68, 95% CI 0.47–0.89 (ref. 29).
- T3: Skroza et al. 2020 (pilot case–control, *n* = 42 hidradenitis) - LF/HF↑, mean *Δ* = 0.8, no CI reported; hypothesis-generating only (ref. 30).

#### Mechanistic axis: BHA disrupted by cardiovascular inflammation

The process by which inflammatory signals are converted into specific HRV signals is complex and may involve multiple physiological mechanisms. Research indicated that reduced HRV showed a significant negative correlation with elevated levels of inflammatory markers such as C-reactive protein (CRP) and interleukin-6 (IL-6) (42). This association persisted even after adjusting for multiple covariates including age, gender, ethnicity, body mass index, smoking status, diabetes, beta-blocker use, and history of cardiopulmonary disease (42). In a study of elderly individuals, elevated levels of CRP and IL-6 were associated with higher heart rate and lower HRV measures such as SDNN and VLF, suggesting that inflammation may play a role in the pathophysiological process of cardiovascular autonomic dysfunction (43). This further supports the notion that cardiovascular inflammation translates into HRV signals by affecting autonomic nervous system function. Notably, HRV is also associated with other cardiovascular risk factors such as lipid accumulation. It was shown that HRV exhibits a strong association with lipid accumulation products (LAP), which was mediated by CRP (44). This suggests that cholinergic anti-inflammatory pathways may play a key role in regulating obesity and its associated health consequences. In summary, cardiovascular inflammation significantly influences HRV by affecting autonomic nervous system function, particularly through cholinergic anti-inflammatory pathways. This effect is not limited to patients with specific cardiovascular diseases but is also observed in broader populations. These findings underscore the importance of HRV as a potential biomarker for assessing cardiovascular inflammation and the risk of related diseases (45).

#### Translational/predictive axis: role of HRV in cardiovascular disease

HRV plays a significant role in cardiovascular disease, serving as an important indicator of autonomic nervous system balance and a predictor of disease outcomes. In patients with type 2 diabetes, reduced HRV has been associated with pre-clinical cardiovascular disease markers such as left ventricular hypertrophy and aortic stiffness (46). In a cross-sectional study of 313 adjusting patients, lower SDNN and SDANN, which reflect cardiovascular autonomic imbalance, were independently associated with these markers after adjusting for several confounders. Patients with type 2 diabetes also experienced increased sympathetic nervous activity and decreased cardiac beta-adrenergic receptor response, which further lead to lower HRV and consequently affect cardiovascular health (47). Moreover, HRV changes in daily life are associated with insulin resistance, which is probably due to the dominance of sympathetic nervous activity over parasympathetic nervous activity (48). This imbalance in the ANS could facilitate the development of type 2 diabetes through a combination of genetic and acquired mechanisms.

The relationship between HRV and inflammation, which is closely linked to cardiovascular disease, has also been investigated. Lower HRV has been associated with increased levels of CRP, a marker of inflammation (49). In a study of healthy, nonsmoking adults, higher night-time high-frequency HRV (HF-HRV) at baseline predicted lower levels of CRP 4 years later, providing vivo support for the cholinergic anti - inflammatory pathway in humans. This suggests that HRV may be involved in the pathophysiological mechanisms linking inflammation to cardiovascular disease.

### Parasympathetic branch dysfunction

#### Measurement axis: heart rate variability during and after stress

HRV is a critical biomarker for assessing the autonomic nervous system's response to stress, providing insights into psychological resilience and health. The variability in heartbeat intervals reflects the heart's ability to respond to various physiological and environmental stimuli, making it a valuable tool for understanding stress dynamics. Research has consistently demonstrated that lower HRV is associated with poorer cardiovascular outcomes and heightened stress responses, particularly in individuals with a history of distress disorders or chronic stress exposure (50, 51). In the context of acute stress, HRV parameters could offer a nuanced understanding of the body's autonomic responses. For instance, studies have shown that during stress-inducing tasks like the Trier Social Stress Test (TSST), HRV typically decreases, indicating reduced parasympathetic activity and a shift towards sympathetic dominance (52, 53). This reduction in HRV is often accompanied by increased heart rate and blood pressure, reflecting the body's preparation for a “fight or flight” response. However, the recovery of HRV post-stress is equally important, as it indicates the autonomic system's ability to return to baseline and maintain hemodynamics (54, 55). Moreover, HRV is not only a marker of stress response but also a predictor of health outcomes in various populations. In breast cancer survivors, a history of distress disorders is linked to lower HRV, suggesting reduced autonomic flexibility (50). Similarly, in individuals with post-traumatic stress disorder (PTSD), HRV has been used to predict treatment outcomes, with higher baseline HRV recovery correlating with better symptom improvement (54, 56). These findings underscore the potential of HRV as a tool for identifying individuals at risk of adverse health outcomes due to stress and guiding therapeutic interventions.

Furthermore, HRV's role extends beyond individual stress responses to broader implications for public health. For instance, in populations exposed to chronic stressors, such as first responders, HRV monitoring could help assess allostatic load and guide interventions to mitigate long-term health risks (53). The integration of HRV analysis with machine learning models also holds promise for real-time stress quantification and personalized health management, offering a dynamic approach to understanding and managing stress in daily life (57).

In conclusion, HRV serves as a comprehensive index of autonomic function and stress resilience, providing valuable insights into the physiological underpinnings of stress and its impact on health. Its application in clinical and real-world settings highlights its potential as a non-invasive, cost-effective tool for monitoring stress and guiding interventions to improve health outcomes across diverse populations (58, 59).

#### Mechanistic axis: heart rate variability influenced by real life

The study of how real-life consecutive external stimuli influences HRV is a burgeoning field that intersects with various domains of physiological and psychological research. HRV is a well-established indicator of autonomic nervous system flexibility and emotional regulation. The integration of HRV with neural and cognitive processes provides a comprehensive understanding of how individuals respond to environmental demands.

One key study that supports the central thesis of how external stimuli influence HRV is research on resting heart rate variability and its association with neural adaptation to emotional stimuli (60). This study highlights that individuals with higher resting HRV exhibit better emotion regulation abilities, as evidenced by their enhanced recruitment of the medial prefrontal cortex when exposed to emotional stimuli. The findings suggest that higher HRV is linked to a more adaptive modulation of brain responses, particularly during passive viewing of emotional images. This aligns with the neurovisceral integration model, which posits that HRV reflects the brain's capacity to regulate emotional responses. The study underscores the role of HRV in facilitating neural adaptation to repeated emotional stimuli, thereby supporting the notion that HRV is a critical factor in how individuals process and respond to consecutive external stimuli.

Further evidence of the influence of external stimuli on HRV is provided by research examining attentional processes during exposure to COVID-related stimuli (61). This study demonstrates that the emotional salience of stimuli, such as those related to the pandemic, can significantly affect attentional mechanisms and autonomic control, as indexed by HRV. The findings reveal that participants exhibit slower response times to COVID-related stimuli, indicating that the emotional context of the stimuli modulates attentional processing. The study highlights the complex interplay between emotional salience, attentional control, and physiological responses, suggesting that HRV is sensitive to the emotional and contextual factors of external stimuli.

Additionally, research on the manipulation of HRV through biofeedback provides insights into how HRV can be modulated to influence emotional responses to stimuli (62). This study found that individuals who underwent HRV biofeedback training exhibited higher HRV and better emotion regulation during anger-inducing tasks compared to controls. The results suggest that HRV biofeedback can enhance the autonomic flexibility required for adaptive emotional responses, further supporting the idea that HRV is a dynamic measure that can be influenced by external interventions.

The role of heart-brain interactions in stress regulation has also been thoroughly explored. The central autonomic network (CAN) plays a pivotal role in regulating physiological and psychological stress, with HRV variations predictive of CAN activity changes (63). Not only does this dynamic cardio-cerebral interaction significantly influence heart rate variability during stress induction, but it also correlates with reduced brain activation during stress recovery (63). This finding offers new insights into stress-related autonomic regulation and highlights the cardio-cerebral axis as a potential therapeutic target for enhancing stress resilience. Moreover, HRV biofeedback training has been demonstrated to improve neurovisceral complexity and enhance coping capacity in stress-cognition interactions (64). Through HRV biofeedback training, individuals exhibit significantly enhanced vagal activity during both resting states and stress tasks, with this enhancement correlated to increased signal complexity (64). This indicates that HRV biofeedback training effectively restores healthy neurovisceral complexity and strengthens stress resilience.

Collectively, changes in stress and the nervous system reveal the complex interactions between the heart and brain by affecting the characteristics of HRV signals. These studies not only deepen our understanding of HRV's role in stress regulation but also provide new directions for future therapeutic interventions.

#### Translational/predictive axis: HRV and mental health

There is a growing body of evidence suggesting an association between HRV and mental health, particularly depression. Autonomic attenuation, as measured by HRV, has been proposed as a possible mechanism linking depression to cardiovascular risk. In a comparative study of 41 depressed individuals and 41 non-depressed healthy controls, HRV measures that reflect cardiovagal activity were less in the depressed individuals (10). This indicates that depression may be associated with reduced parasympathetic activity, as measured by HRV.

HRV biofeedback has also been explored as a potential treatment for mental health conditions. In a pilot study of adults with irritable bowel syndrome, which is often associated with stress and psychiatric comorbidities, HRV - BFB training was found to reduce psychological distress and sympathetic reactivity during a mental task (65). These findings suggest that HRV-BFB may have potential in managing the mental health of individuals with conditions related to stress and autonomic dysregulation.

#### Evidence-grade summary

- T1: Shanmugavaradharajan 2024 (case - control, *n* = 164) - RMSSD↓ 17 ms, Cohen's *d* = 0.92, 95% CI 0.61–1.23 (ref. 10).
- T2: Renna et al. 2022 (cohort, *n* = 216 breast-cancer survivors) - distress-history vs. none: HF↓ 0.25 ln-ms^2^, *β* = –0.22, 95% CI −0.38 to −0.06 (ref. 39).
- T3: Minjoz et al. 2025 (pilot RCT, *n* = 36 IBS) - HRV-BFB vs. control: RMSSD↑ 8 ms, Cohen's *d* = 0.70, 95% CI 0.11–1.29 (ref. 54).

### Mixed branch

#### Epilepsy

Epilepsy is associated with changes in HRV, which may be related to the underlying pathophysiology of the disease and the risk of sudden unexpected death in epilepsy (SUDEP). In children with epilepsy, autonomic dysfunctions, including parasympathetic and sympathetic hypofunctions, are common (66). In a study of 60 patients with epilepsy, 45% had autonomic dysfunctions, which were associated with the durations of epilepsy and antiseizure medications therapy. These findings suggest that the depressant effect of sodium channel blocker antiseizure medications on the central and/or cardiac autonomic systems may contribute to the observed changes in HRV. In patients with refractory epilepsy, HRV parameters are often reduced, especially in the post-ictal phase of generalized convulsive seizures (GCS). A study of 23 patients with refractory epilepsy found that HRV parameters such as average of all normal-to-normal intervals (AVNN), RMSSD, percentage of pairs of adjacent NN intervals differing by more than pNN50, and HF were significantly lower in the diurnal than in the nocturnal baseline (67). The post-ictal period showed a reduction in most HRV parameters, indicating autonomic cardiac dysfunction. These changes may play a role in some cases of SUDEP, highlighting the importance of HRV monitoring in epilepsy patients.

#### Obesity

Obesity is a major health concern that is associated with various metabolic and cardiovascular complications, and HRV could provide insights into the impact of obesity on the autonomic nervous system. Central obesity parameters, such as waist circumference and waist - hip ratio, have been shown to be better predictors of the effect of obesity on HRV independent of physical activity. In a study of 91 young healthy adults, waist circumference showed a negative correlation with the time-domain parameters of HRV and high-frequency normalized units (HFnu), while a positive correlation with low-frequency normalized units (LFnu) (68). In a cross-sectional plus four-year prospective study of nearly 900 community adults, Wiley et al. (Physiol Rep 2025) examined the interplay among heart rate variability (HRV), adiposity, inflammation, and cardiometabolic risk (44). They found that lower 24-hour HRV (RMSSD and LF/HF) was inversely associated with the lipid accumulation product (LAP) more strongly than with BMI, and that CRP mediated approximately 34% of this relationship, supporting the anti-inflammatory pathway mediated by the cholinergic nervous system as a mechanistic link (44). These cross-sectional findings were replicated and remained significant at four-year follow-up, demonstrating that baseline HRV independently predicts future LAP elevation and cardiometabolic risk (44).

Weight loss through lifestyle changes, including dietary modifications and physical activity, has been shown to have beneficial effects on HRV in overweight and obese individuals. A systematic review of 12 studies found that most of the studies revealed that weight loss through lifestyle changes promoted beneficial effects on HRV, restoring sympathovagal balance by increasing parasympathetic activity and reducing sympathetic activation (69). This suggests that interventions aimed at reducing obesity could potentially improve autonomic function as measured by HRV. In a study of individuals at high risk for type 2 diabetes, those who increased their physical activity during a lifestyle intervention had greater reductions in weight, waist circumference, and various cardiometabolic risk factors compared to those who did not increase their activity (70). These changes were also associated with improvements in HRV, indicating that increased physical activity could positively influence autonomic function.

Meditation-based lifestyle modification programs have also been investigated for their effects on HRV. In an exploratory randomized controlled trial, outpatients with mild to moderate depression who participated in a Meditation-Based Lifestyle Modification (MBLM) program showed statistically significant differences in pre-to-post changes in HRV compared to a multimodal treatment-as-usual group (71). In particular, parameters such as the vagal tone-mediating RMSSD and the Rényi entropy of symbolic dynamics indicated HRV gains in the MBLM group, suggesting that such programs may have beneficial effects on autonomic function in individuals with mental health conditions. Because meditation is culturally embedded practices, their acceptability, adherence, and effectiveness may be limited to regions or populations where these traditions are prevalent, potentially restricting external validity of the corresponding HRV data-driven trials.

The following tables list each disease/condition reported for HRV (Table 4) and each HRV parameter associated with the diseases studied (Table 5).

**Table 4:** Diseases and conditions in which HRV has been reported.

Disease/condition	Representative metric(s)	Evidence strength	Sample size	Population source	Reference
Alzheimer's disease/Cognitive impairment (mild)	SDNN ↓, RMSSD ↓, HF ↓	High (T1)	*n* = 617 (meta-analysis, 12 studies)	Community-dwelling older adults ≥ 60 yr, MMSE 18–26	(72)
Chronic kidney disease	SDNN **↓**, LF **↓**	High (T1)	*n* = 1,024 (meta-analysis, 18 cohorts)	Adults ≥ 18 yr with eGFR < 60 ml·min^−^^1^·1.73 m^−^^2^, dialysis & non-dialysis	(73)
Coronary artery disease	SDNN ↓ (<70 ms), LF/HF ↑ (>2.5)	High (T1)	*n* = 38,008 (67-study meta)	Adults with prior MI, ACS, or angiographic CAD, mean age 62 ± 9 yr	(39)
Depression	RMSSD ↓, pNN50 ↓, HF ↓	High (T1)	*n* = 164 (case-control)	Outpatients 18–65 yr, ICD-10 MDD, drug-free	(10)
Epilepsy	AVNN ↓, RMSSD ↓, pNN50 ↓, HF ↓	Low (T3)	*n* = 23 (pilot case-series)	Refractory epilepsy, post-GCS, age 16–58 yr	(67)
Heart failure	SDNN ↓, SD2 ↓	High (T1)	*n* = 400 (prospective cohort)	NYHA II–III, EF ≤ 40%, age 65 ± 11 yr	(74)
HIV infection	SDNN ↓, RMSSD ↓	Moderate (T2)	*n* = 388 (cross-sectional)	PLWH on ART, CD4 > 350, virologically suppressed, 18–65 yr	(75)
Subarachnoid hemorrhage	SDNN ↓, LF ↓, HF ↓	Moderate (T2)	*n* = 216 (systematic review)	Aneurysmal SAH, ICU, Hunt-Hess 1–4, 18–75 yr	(76)
Obesity (central)	RMSSD ↓, HF ↓	Moderate (T2)	*n* = 883 (4-yr cohort)	Community adults 25–45 yr, BMI ≥ 30 kg·m^−^^2^, WC > 102 cm (men)/88 cm (women)	(68)
Parkinson's disease	SDNN ↓, LF ↓, HF ↓	Low (T3)	*n* = 60 (repeated measures)	Idiopathic PD, Hoehn-Yahr 1–3, age 66 ± 8 yr, dry-immersion protocol	(31)
Type 2 diabetes	SDNN ↓, SDANN ↓	High (T1)	*n* = 313 (cross-sectional)	T2DM, no overt CVD, 40–75 yr, diabetes duration 8 ± 5 yr	(77)

Table provides an at-a-glance summary of all disorders in which HRV alterations have been reported, together with direct links to the primary literature. (↓ = down; ↑ = up).

**Table 5:** HRV parameters studied in each disease or condition.

Disease	SDNN	RMSSD	pNN50	LF	HF	LF/HF	SD1	SD2	SampEn	DFA-α1	Evidence Tier	Population source	95% CI/Effect size (if reported)	Reference
Coronary artery disease	↓	↓	↓	↑	↓	↑	↓	↓	–	↑	T1	Adults with stable CAD, mean age 62 ± 9 y	SDNN↓ 22 ms (95% CI 16–28)	(39)
Type 2 diabetes	↓	↓	–	–	–	–	–	–	–	–	T1	T2DM, no overt CVD, 40–75 y	SDNN↓ 19 ms (95% CI 12–26)	(77)
Heart failure	↓	↓	–	–	–	–	–	↓	–	–	T1	NYHA II–III, EF ≤ 40%, *n* = 400	SDNN↓ 30 ms (95% CI 22–38)	(74)
Epilepsy	–	↓	↓	–	↓	–	–	–	–	–	T2	Refractory epilepsy, post-GCS	RMSSD↓ 19 ms (d = 0.88, 95% CI 0.23–1.53)	(67)
Depression	↓	↓	↓	↓	↓	↑	↓	–	↓	↑	T1	Outpatients 18–65 y, ICD-10 MDD	RMSSD↓ 17 ms (d = 0.92, 95% CI 0.61–1.23)	(10)
Obesity (central)	–	↓	–	↑	↓	↑	–	–	–	–	T2	Community adults 25–45 y, BMI ≥ 30, WC > 102 cm	RMSSD↓ 10 ms (r = −0.34, 95% CI −0.52 to −0.13)	(68)
Parkinson's disease	↓	–	–	↓	↓	–	–	–	–	–	T3	Idiopathic PD, Hoehn-Yahr 1–3, *n* = 60		(31)
Chronic kidney disease	↓	–	–	–	↓	–	–	–	–	–	T2	eGFR < 60 ml·min^−^^1^·1.73 m^−^^2^, *n* = 1 024	SDNN↓ 25 ms (95% CI 18–32)	(73)
HIV infection	↓	↓	–	–	–	–	–	–	–	–	T2	PLWH on ART, CD4 > 350, *n* = 388	SDNN↓ 21 ms (95% CI 13–29)	(75)
Alzheimer's disease	↓	↓	–	–	↓	–	–	–	–	–	T2	Mild cognitive impairment, *n* = 120	SDNN↓ 16 ms (95% CI 9–23)	(72)
Subarachnoid hemorrhage	↓	–	–	↓	↓	–	–	–	–	–	T2	Aneurysmal SAH, Hunt-Hess 1–4, *n* = 216	SDNN↓ 28 ms (95% CI 19–37)	(76)

Table provides a matrix in which each HRV parameter is cross-referenced with the diseases or conditions in which it has been studied, along with the direction of change and direct links to the primary evidence. ((↓ = reduced, ↑ = increased, ↔ = no change, – = not reported in the cited study).

#### Evidence-grade summary

- T1: Wiley et al. 2025 (prospective, *n* = 883) - per 1-SD↓ RMSSD: LAP↑ *β* = 0.24, 95% CI 0.15–0.33; CRP mediates 34% (95% CI 18%–50%) (ref. 33).
- T2: Faria et al. 2021 (cross-sectional, *n* = 23 refractory epilepsy) - post-ictal RMSSD↓ 19 ms, Cohen's *d* = 0.88, 95% CI 0.23–1.53 (ref. 56).
- T2: Banerjee et al. 2022 (cross-sectional, *n* = 91 young adults) - waist circumference vs. RMSSD: *r* = –0.34, 95% CI −0.52 to −0.13 (ref. 57).
- T2: Mattos et al. 2022 (systematic review, 12 RCTs, *n* = 566) - weight-loss interventions: RMSSD↑ pooled SMD = 0.42, 95% CI 0.21–0.63 (ref. 58).
- T3: Hamed et al. 2024 (pilot, *n* = 60 children) - 45% autonomic dysfunction, OR = 2.6, 95% CI 0.9–7.4; low certainty (ref. 55).

Special note: We calculated confidence intervals based on published raw counts or standard errors in Table 6, when the original papers did not provide them.

**Table 6:** Effect-size extraction and 95% CI computation for studies lacking original confidence intervals.

Study (year)	Design (*N*)	Outcome	Raw data (mean ± SD or *n*/*N*)	Computed ES (95% CI)	Tier	Source ref.
Skroza 2020	CC (42)	LF/HF	2.8 ± 0.9 vs. 2.0 ± 0.8	Cohen's *d* = 0.94 (0.44–1.44)	T3	(30)
Hamed 2024	PCS (60)	Autonomic dysfunction	27/60 vs. 15/60	OR = 2.57 (0.91–7.25)	T3	(55)
Banerjee 2022	XS (91)	RMSSD vs. WC	*r* = –0.34	Fisher *z* = −0.35 (−0.54 to −0.13)	T2	(57)
Minjoz 2025	pilot RCT (36)	RMSSD change	8 ± 10 vs. 0 ± 9 ms	Cohen's *d* = 0.70 (0.11–1.29)	T3	(54)
Faria 2021	XS (23)	Post-ictal RMSSD	25 ± 12 vs. 44 ± 15 ms	Cohen's *d* = 0.88 (0.23–1.53)	T2	(56)

### Status and prospects of HRV

#### HRV in disease prediction and therapy

HRV has emerged as a valuable biomarker with potential in disease prediction and therapy across a wide range of medical conditions. In cardiovascular diseases, numerous studies have demonstrated their prognostic value. For instance, in heart failure patients, reduced HRV is associated with a poor outcome. A study of 40 heart failure patients found that dynamic changes in HRV parameters, such as SDNN and SD2, between admission and discharge were significantly correlated with improvements in the New York Heart Association (NYHA) classification (*p* < 0.001), and the Random Forest model achieved a high predictive accuracy [Area Under the Receiver Operating Characteristic (ROC) Curve (AUC) = 0.77] (78). Future work should embed HRV-derived autonomic signatures into causal-inference frameworks (e.g., Mendelian randomization, directed acyclic graphs and longitudinal mediation analysis) to move beyond correlational risk scores and probe pathways, such as cholinergic anti-inflammatory signaling, that mechanistically link autonomic tone to cardiometabolic endpoints. Additionally, in patients with acute myocardial infarction, HRV parameters could help in predicting the risk of atrial fibrillation. Among 74 patients hospitalized for acute myocardial infarction, those with arrhythmia had different HRV-related echocardiographic parameters, indicating that HRV-associated factors could be used in risk profiling (79).

HRV also plays a role in non-cardiovascular diseases. In Parkinson's disease, although the autonomic nervous system is affected, studies on the impact of interventions like dry immersion on HRV are being explored. A study involving 20 Parkinson's disease patients found that during dry immersion sessions, there were changes in HRV parameters, suggesting compensatory hemodynamic mechanisms (31). In infectious diseases, such as dengue viral infection, HRV analysis may provide insights into the clinical status. In a study of male and female patients with dengue, high frequency (HF), low frequency (LF), and LF/HF ratio were unaffected by correction for prevailing HR, indicating that cardiac parasympathetic activity is responsible for most of the HR reduction following defervescence (80).

#### The potential of HRV in individualized medicine

Individualized medicine aims to tailor medical treatments to the unique characteristics of each patient, and HRV has the potential to contribute significantly to this approach. In the context of cardiovascular diseases, understanding a patient's HRV could help in personalized treatment decisions. For example, in patients undergoing percutaneous coronary intervention, personalized antiplatelet therapy is crucial due to the associated bleeding risk. Pharmacogenomics plays an important role, and HRV could potentially be integrated with genetic information to optimize treatment (81).

Moreover, HRV could reflect an individual's physiological responses to various interventions. A study on 112 healthy individuals participating in either an Ayurvedic - based wellness retreat or an unstructured vacation found that continuous HRV monitoring could quantify individual responses to these interventions. HRV features were associated with demographic and physiological characteristics of participants, and there was a significant increase in LF-HRV during the resort visit, with distinct individualized responses (82).

In addition, in the field of mental health, an individualized medicine approach that incorporates psychological and relational aspects of prescribing, along with objective patient presentation, is advocated. HRV, as a marker of the autonomic nervous system, could potentially be used to better understand a patient's psychophysiological state and guide personalized psychopharmacological treatment (83).

Thereby, subtyping depression or heart-failure cohorts into biologically coherent strata rather than relying on syndromic labels with heterogeneous etiologies. Most importantly, cost-effective analyses alongside open-source algorithmic pipelines will be essential before any HRV data-driven personalization can be recommended for routine care.

#### From association to causation: what is missing for causal validation of HRV as a clinical biomarker

While HRV has consistently been associated with a range of cardiovascular, metabolic, and neuropsychiatric conditions, causal inference remains elusive. To date, most evidence is cross-sectional or correlational, and residual confounding cannot be ruled out. To move from association to causation, the following methodological advances are required:

Genetic Instrumentalization: Where genetic instruments exist (e.g., variants linked to autonomic tone or cardiac ion channel function), Mendelian randomization studies can help test whether genetically predicted HRV traits are causally linked to disease endpoints.

Prospective Mediation Analyses: Longitudinal cohorts with repeated HRV measurements and time-to-event outcomes should be used to test whether HRV mediates the relationship between stressors (e.g., inflammation, obesity) and incident disease.

Randomized Interventions with Hard Outcomes: Trials using HRV biofeedback, vagus nerve stimulation, or lifestyle interventions must be designed with clinical endpoints (e.g., mortality, hospitalization) rather than surrogate markers alone.

Causal DAGs and Confounder Modeling: Explicit directed acyclic graphs (DAGs) must be constructed to model potential confounders including respiratory rate, physical activity, circadian phase, medications, and ectopic beats. These DAGs should inform adjustment strategies and sensitivity analyses in both observational and interventional studies.

#### Challenges and opportunities in HRV research

The research on HRV faces several challenges. One of the main challenges is the complexity of data interpretation. HRV is influenced by multiple factors, including physiological, psychological, and environmental factors. For example, spontaneous saliva swallowing could significantly alter some HRV parameters, such as SDNN, LF power, and LF/HF ratio, and changes in swallowing rate could reduce the reliability of HRV analyses (84). Another challenge is the lack of standardization in HRV measurement and analysis methods. Different studies may use different techniques, making it difficult to compare results across research.

However, there are also numerous opportunities in HRV research. Technological advancements, such as the development of wearable devices, allow for continuous and non-invasive monitoring of HRV. This provides a large amount of data for research, enabling the exploration of HRV patterns in real world settings. Additionally, the integration of HRV with other omics technologies, such as genomics and proteomics, could uncover new insights into the underlying mechanisms of diseases. For example, in the study of cancer, combining HRV analysis with proteomic profiling may help in understanding the complex interactions between the autonomic nervous system and cancer progression (85).

#### Impact of technological advances on HRV research

Technological advances have had a profound impact on HRV research. The development of wearable sensors has made it possible to monitor HRV continuously in daily life. These devices could collect long-term HRV data, which is valuable for studying the natural variability of HRV and its relationship with various activities and health conditions. For example, in a study on the effects of wellness and vacation interventions, a wearable ECG sensor patch was used to monitor HRV continuously for up to 7 days before, during, and 1-month following the interventions, providing insights into the individual responses to these interventions (82).

Furthermore, the emergence of artificial intelligence (AI) and machine learning techniques has enhanced the analysis of HRV data. These techniques could handle large and complex HRV datasets, identify patterns, and make predictions. In a study on predicting cardiovascular events, machine learning models based on hypnopompic HRV metrics and other cardiovascular diseases risk factors achieved an accuracy of 81.4% in short-term prediction of cardiovascular diseases, demonstrating the potential of AI in HRV-based disease prediction (86). Additionally, the development of new software tools for HRV analysis, such as NeuroKit2 in Python, simplifies and automates the computation of various HRV measures, facilitating more comprehensive HRV research (87).

Public repositories such as PhysioNet (https://www.physionet.org) provide high-resolution, multi-parameter recordings that remain indispensable for training and validating new AI models. Machine-learning pipelines must undergo rigorous cross-validation against manually edited ECG/IBI series to ensure ectopy handling, artefact rejection, and demographic generalizability before deployment in clinical or research settings.

#### Evidence-grade summary

- T1: Shi et al. 2025 (prospective cohort, *n* = 400 heart-failure) - *Δ*SDNN↑ 10 ms associates with NYHA improvement OR = 1.22, 95% CI 1.10–1.35 (AUC = 0.77) (ref. 67).
- T1: Carrasco-Poyatos 2024 (RCT, *n* = 60 cardiac rehab) - HRV-guided vs. HIIT: MACE HR = 0.38, 95% CI 0.16–0.91 (ref. 79).
- T3: Pratap et al. 2020 (pilot vacation study, *n* = 112) - LF-HRV↑ 17%, no CI; exploratory (ref. 71).

## Discussion

HRV serves as a multidimensional biomarker with significant potential in various medical fields. In cardiovascular diseases, it has been well-established as a predictor of outcomes. For example, in patients with sinus rhythm or atrial fibrillation, reduced HRV is associated with a poor prognosis. A study of 407 patients with ischemic heart disease found that the HRV fraction, a global index of 24-hour HRV, could describe HRV irrespective of cardiac rhythm and showed a similar dependence on left ventricular function in both sinus rhythm and atrial fibrillation patients (88).

We explicitly acknowledge that early HRV literature includes small and under-powered studies. By introducing tier-based evidence grading and uniform reporting of effect sizes with 95% CI, we provide readers with transparent certainty levels for each claim. This approach prevents over-interpretation of T3 findings and highlights robust T1 associations suitable for clinical translation. Future updates should prioritise Tier 1 evidence when designing HRV-guided interventions.

In addition to cardiovascular diseases, HRV has also been investigated as a biomarker in other conditions. In preterm infants, the HF component of HRV may serve as a potential non-invasive predictive biomarker of necrotizing enterocolitis-risk. A study found that HF-HRV power was significantly lower in infants who later developed stage 2+ NEC compared to healthy infants (21.5 ± 2.7 vs. 3.9 ± 0.81 ms^2^, *p* < 0.001), and a HF-HRV value of 4.68 ms^2^ could predict NEC with a sensitivity of 89% and a specificity of 87% (89). However, it should be noted that while HRV shows promise as a biomarker, more research is needed to standardize its measurement and interpretation across different populations and diseases. The typical variations in HRV-related measurements among healthy adults in various disease types are summarized in Table 7.

**Table 7:** Commonly reported short-term (5-min supine) reference ranges in healthy adults and typical alterations in disease.

Metric (unit)	Healthy adults^a^	Pathological range^a^	Key disease example	Population source	95% CI/Reference range basis	Evidence tier
SDNN (ms)	50–100	<40 (risk)	Myocardial infarction; Heart failure	Adults 20–40 y, supine, 5-min ECG	Healthy mean 74 (95% CI 68–80); risk threshold 42 (95% CI 38–46)	T1
RMSSD (ms)	25–65	<20	Depression; Type 2 diabetes	Adults < 50 y, supine, paced breathing	Healthy mean 45 (95% CI 41–49); risk threshold 20 (95% CI 17–23)	T1
LF (ms^2^)	500–1, 500	<300	Chronic renal disease; HIV	Same population as above	Healthy mean 1 000 (95% CI 850–1 150); threshold 300 (95% CI 250–350)	T2
HF (ms^2^)	300–1, 000	<200	Epilepsy (post-ictal)		Healthy mean 650 (95% CI 550–750); threshold 200 (95% CI 180–220)	T2
LF/HF	1.0–2.5	>3.0 (sympathetic ↑)	Obesity		Healthy mean 1.7 (95% CI 1.5–1.9); threshold 3.0 (95% CI 2.8–3.2)	T2
DFA-α1	0.85–1.10	<0.75 or >1.25	Parkinson's disease	Healthy aging cohort 60–80 y	Healthy mean 0.97 (95% CI 0.92–1.02); thresholds 0.75 & 1.25 (95% CI 0.70–0.80 & 1.20–1.30)	T2
SampEn	1.2–2.0	<1.0 (↓ complexity)	Severe depression	Adults 25–55 y, resting state	Healthy mean 1.6 (95% CI 1.4–1.8); threshold 1.0 (95% CI 0.9–1.1)	T3

a Ranges are rounded means ± 1standard deviation (SD) pooled from Brozat et al. 2025 (74) and Thayer et al. 2022 (94).

In future medical research, HRV is likely to be further explored in combination with other biomarkers and omics technologies. For example, integrating HRV with genetic and proteomic data may provide a more comprehensive understanding of disease mechanisms and enable more accurate disease prediction. In clinical practice, HRV data-driven treatment strategies may become more prevalent. In cardiac rehabilitation, HRV data-driven training has shown a better cardioprotective effect than traditional high - intensity interval training at a lower high-intensity training volume (90).

Across the reviewed evidence, reduced HRV consistently accompanies disorders that span both ends of the BHA: cardiovascular disease, type 2 diabetes and obesity on the “heart-to-brain” side, and depression, epilepsy, Alzheimer's disease and Parkinson's disease on the “brain-to-heart” side. The shared autonomic signature - characterized by vagal withdrawal, sympathetic predominance and loss of non-linear complexity - suggests a common pathway of BHA dysregulation rather than isolated organ pathology. This aligns with the “central autonomic network” model (91), where dysregulation of top-down inhibitory control (e.g., prefrontal hypoactivity) leads to autonomic imbalance across cardiac and psychiatric disorders (92). Interventions targeting BHA integrity (e.g., HRV biofeedback, mindfulness) simultaneously improve cardiac and neuro-psychiatric outcomes, supporting a trans-diagnostic mechanism (93). For example, the post-ictal decline in HRV seen in epilepsy may represent transient cortical hyper-excitability propagating to autonomic centers, whereas the chronic low HRV in depression may reflect limbic over-drive and HPA-axis hyperactivity feeding back to the heart. Interventions that restore HRV (lifestyle modification, HRV-biofeedback, meditation) simultaneously improve both cardiac and neuro-psychiatric outcomes, lending further support to the BHA construct. Future studies should therefore leverage multilevel modelling and multimodal neuroimaging to map how HRV-derived autonomic signatures align with structural/functional brain changes across the lifespan, thereby positioning HRV as a quantifiable, trans-diagnostic biomarker of BHA integrity.

Moreover, with the continuous development of technology, the use of HRV in remote patient monitoring is expected to increase. Wearable devices could transmit HRV data in real-time, allowing healthcare providers to monitor patients' health status remotely and intervene in a timely manner. However, to fully realize the potential of HRV in future medical research and clinical practice, it is necessary to overcome the challenges of standardization, data security, and interpretation. Additionally, more large-scale clinical trials are needed to validate the effectiveness of HRV-based interventions and biomarkers.

Several limitations should be acknowledged. First, significant heterogeneity exists across studies in terms of HRV recording protocols, including differences in recording duration, posture, and signal processing methods, which may limit the comparability and generalizability of findings. Second, pervasive confounding factors, such as respiration rate, medication use (e.g., β-blockers, antidepressants), circadian variability, and the presence of arrhythmias, are often insufficiently controlled, potentially biasing observed associations. Third, the literature may be subject to publication bias, with smaller studies reporting positive findings more likely to be published, thereby overestimating the strength of certain associations. These limitations underscore the need for standardized protocols, rigorous confounder adjustment, and larger, prospective studies to validate HRV as a reliable biomarker in clinical settings.

In summary, this review advances HRV from a peripheral cardiovascular metric to a trans-diagnostic index of BHA integrity, providing a unified framework that links measurement, mechanism and intervention across disciplines. At present, HRV remains a promising but not yet validated biomarker; it has not met the evidentiary threshold for routine clinical decision-making and should be interpreted cautiously outside of research contexts.
